# Supplementary material for: Polyketide synthase gene clusters in the ericoid mycorrhizal fungus Oidiodendron maius: genome-wide prediction, expression profiling, and functional characterization of knockout mutants
Source: Front Plant Sci. 2026 Sep 16;17:1924430. doi: 10.3389/fpls.2026.1924430 (PMC13623903; doi:10.3389/fpls.2026.1924430)
Supplement: Supplementary file 1 [file SupplementaryFile1.zip › Supplementary Materials/Supplementary Folder 1.zip!/Oidma1_AssemblyScaffolds_Repeatmasked/knownclusterblast/cluster56/jgi.p_Oidma1_46521_mibig_hits.html]

| MIBiG Protein | Description | MIBiG Cluster | MiBiG Product | % ID | % Coverage | BLAST Score | E-value |
| --- | --- | --- | --- | --- | --- | --- | --- |
| CBX55165.1 | P450\_monooxygenase | BGC0001243 | Botryenalol\_/\_botrydial\_/\_dihydrobotrydial\_/\_botryendial\_/ | 35 | 105.148514851 | 289 | 6.1e-78 |
| EAU36745.1 | hypothetical\_protein | BGC0000292 | Acetylaranotin\_biosynthetic\_gene\_cluster | 35 | 97.2277227723 | 266 | 5.5e-71 |
| CAA75567.1 | cytochrome\_P450\_monooxygenase | BGC0001270 | Gibberellin\_A3\_/\_gibberellin\_A1\_/\_gibberellin\_A7\_/\_gibberellin\_A4\_/ | 39 | 74.6534653465 | 235 | 1.8e-61 |
| CAP07652.1 | P450\_monooxygenase | BGC0001269\_c4 | Gibberellin\_A3\_/\_gibberellin\_A1\_/\_gibberellin\_A7\_/\_gibberellin\_A4\_/ | 30 | 80.198019802 | 194 | 2.7e-49 |
| EBA27371.1 | cytochrome\_P450\_monooxygenase,\_putative | BGC0000129 | Pyripyropene\_biosynthetic\_gene\_cluster | 33 | 77.2277227723 | 192 | 1.3e-48 |
| CAP07651.1 | P450\_monooxygenase | BGC0001269\_c5 | Gibberellin\_A3\_/\_gibberellin\_A1\_/\_gibberellin\_A7\_/\_gibberellin\_A4\_/ | 33 | 76.4356435644 | 191 | 2.3e-48 |
| AGO86661.1 | putative\_cytochrome\_p450 | BGC0001255 | Equisetin\_biosynthetic\_gene\_cluster | 33 | 81.9801980198 | 191 | 2.9e-48 |
| BAD29974.1 | P450\_monooxygenase\_2 | BGC0000676 | Aphidicolin\_biosynthetic\_gene\_cluster | 30 | 94.0594059406 | 191 | 2.9e-48 |
| CAA75565.1 | cytochrome\_P450\_monooxygenase | BGC0001270\_c4 | Gibberellin\_A3\_/\_gibberellin\_A1\_/\_gibberellin\_A7\_/\_gibberellin\_A4\_/ | 31 | 95.2475247525 | 188 | 1.5e-47 |
| CAP07653.1 | P450\_monooxygenase | BGC0001269\_c3 | Gibberellin\_A3\_/\_gibberellin\_A1\_/\_gibberellin\_A7\_/\_gibberellin\_A4\_/ | 30 | 94.8514851485 | 183 | 8e-46 |
| CAA75566.1 | cytochrome\_P450\_monooxygenase | BGC0001270\_c2 | Gibberellin\_A3\_/\_gibberellin\_A1\_/\_gibberellin\_A7\_/\_gibberellin\_A4\_/ | 30 | 74.4554455446 | 180 | 4e-45 |
| AAK11528.1 | PaxP | BGC0001082 | Paxilline\_biosynthetic\_gene\_cluster | 30 | 76.4356435644 | 180 | 6.8e-45 |
| BAV32161.1 | cytochrome\_P450\_monooxygenase | BGC0001373 | Sordarin\_biosynthetic\_gene\_cluster | 32 | 73.4653465347 | 178 | 1.5e-44 |
| BAM84045.1 | cytochrome\_P450\_monooxygenase | BGC0001260 | Terpendole\_E\_biosynthetic\_gene\_cluster | 26 | 103.762376238 | 177 | 3.4e-44 |
| EAU29532.1 | conserved\_hypothetical\_protein | BGC0000682 | Terretonin\_biosynthetic\_gene\_cluster | 29 | 79.0099009901 | 170 | 4.1e-42 |
| AAK11527.1 | PaxQ | BGC0001082 | Paxilline\_biosynthetic\_gene\_cluster | 30 | 77.2277227723 | 170 | 7e-42 |
| AGC83579.1 | P450\_monooxygenase | BGC0000818 | Notoamide\_biosynthetic\_gene\_cluster | 31 | 69.900990099 | 165 | 1.7e-40 |
| AAW88512.1 | P450\_monooxygenase | BGC0001081 | Lolitrem\_biosynthetic\_gene\_cluster | 26 | 87.9207920792 | 160 | 7.3e-39 |
| BAM84048.1 | cytochrome\_P450\_monooxygenase | BGC0001260 | Terpendole\_E\_biosynthetic\_gene\_cluster | 30 | 71.0891089109 | 149 | 1.3e-35 |
| CBF87243.1 | cytochrome\_P450,\_putative\_(Eurofung) | BGC0000680\_c2 | Austinol/dehydroaustinol,\_locus\_1\_biosynthetic\_gene\_cluster | 30 | 79.2079207921 | 148 | 1.7e-35 |
| CBF87249.1 | cytochrome\_P450,\_putative\_(Eurofung) | BGC0000680\_c2 | Austinol/dehydroaustinol,\_locus\_1\_biosynthetic\_gene\_cluster | 28 | 79.801980198 | 147 | 3.7e-35 |
| BAE56598.1 |  | BGC0001123 | Aspirochlorine\_biosynthetic\_gene\_cluster | 26 | 95.4455445545 | 146 | 8.3e-35 |
| AGA37282.1 | P450\_monooxygenase | BGC0000819 | Paraherquamide\_biosynthetic\_gene\_cluster | 29 | 63.1683168317 | 139 | 1e-32 |
| ABF20223.1 | P450\_monooxygenase | BGC0001081\_c2 | Lolitrem\_biosynthetic\_gene\_cluster | 26 | 78.6138613861 | 139 | 1e-32 |
| BAC20565.1 | cytochrome\_P450 | BGC0000039 | Compactin\_biosynthetic\_gene\_cluster | 29 | 74.0594059406 | 138 | 2.3e-32 |
| ctg1\_orf003 |  | BGC0001068 | Pyripyropene\_A\_biosynthetic\_gene\_cluster | 39 | 30.495049505 | 104 | 2.8e-22 |
| ctg1\_orf00001 |  | BGC0000685 | Brassicicene\_C\_biosynthetic\_gene\_cluster | 41 | 20.7920792079 | 77 | 3.6e-14 |
| BAE56590.1 |  | BGC0001123 | Aspirochlorine\_biosynthetic\_gene\_cluster | 27 | 22.3762376238 | 50 | 8.1e-06 |
